# Anæsthesia of the Teeth by Means of High-Frequency Currents (D’Arsonval)

**Published:** 1902-09

**Authors:** William H. Potter


### Reviews of Dental Literature.

Anæsthesia of the Teeth by Means of High-Frequency Currents (d'Arsonval). By Dr. Rudolf Bum, Vienna.[1]

The author first refers to the experiments of Professor d'Arsonval in regard to the physiological action of the Tesla high-frequency currents. Such currents have proved to be, in spite of their high tension, entirely harmless. They have for some time been used in electro-therapeutics in the treatment of neuralgias and affections of the skin. It was later demonstrated that such currents when applied to the skin for a sufficient length of time produced complete anæsthesia. In 1893 Drs. Cruet and Oudin, in Paris, used high-frequency currents to produce anæsthesia in tooth-extraction. A moist wad of cotton was placed about the roots of the tooth to be extracted, and this was made one electrode. The other electrode was applied to the outside of the cheek. In about fifty per cent. of the cases reported the results were entirely satisfactory. In spite of these favorable results little was heard of the method until recently. when the work of Drs. L. R. Regnier and H. Didsbury (Paris) became known. These investigators used the current not only for tooth-extraction, but for the control of sensitive dentine. They lay down the following rules of procedure:

1. The mucous membrane before the operation must be washed with alcohol and permanganate of potassium.
2. The operation-table must be free from all metallic substances.

---

[1] Ueber Anästhesie der Zähne mit Hilfe von Hochfrequenzströmen (d'Arsonval), von Dr. Rudolf Bum, Zahnarzt in Wien. Œsterreichisch-ungarishe Vierteljahrsschrift für Zahnheilkunde, April, 1902.

3. A current of one hundred and fifty to three hundred milliampères must be at command.

4. The proper setting up of the apparatus is of the greatest importance.

The results in the hands of these experimenters were surprisingly good. Single-rooted teeth after an application of a current of one hundred and fifty to three milliampères for from three to five minutes could be extracted without pain. In the case of teeth with more than one root, a loss of sensation was reached by the use of a current of two hundred and fifty to five hundred milliampères for from five to eight minutes. The author has undertaken to verify the observations of the above quoted men; and with the aid of Dr. Leopold Freund, of Vienna, and his apparatus has conducted a series of experiments. He finds that the application of a high-frequency current to the skin produces first an anæmia in the form of chalk-white blisters and an erection of the papillæ (goose-flesh). This condition gives place after a few minutes to a hyperæmia with strong erythematous coloring.

Subjectively a certain degree of loss of sensibility appears immediately after the application of the current. This loss of sensation can go on to complete insensibility. The anæsthesia according to the author, is due to a loss of nourishment, and the destruction of function of the sensory nerves due to the anæmia. In his practical cases the author says, "In some cases we had an entirely satisfactory result, the extraction was entirely painless. Other cases gave a negative or, at least, a questionable result. The small amount of bleeding after the extraction was always noticeable. We are to-day only in the position to say that the high-frequency currents possess a decided anæsthetizing effect; but whether this anæsthesia will reach the point desirable for the painless extraction of teeth cannot now be safely said." The author promises another communication at the close of further investigations.

William H. Potter.

